# Dual Role of Endothelial Nitric Oxide Synthase in Oxidized LDL-Induced, p66^Shc^-Mediated Oxidative Stress in Cultured Human Endothelial Cells

**DOI:** 10.1371/journal.pone.0107787

**Published:** 2014-09-23

**Authors:** Yi Shi, Thomas F. Lüscher, Giovanni G. Camici

**Affiliations:** 1 Cardiology, University Heart Center, University Hospital Zürich and Center for Molecular Cardiology, Campus Schlieren, University of Zurich, Zurich, Switzerland; 2 Center for Integrative Human Physiology (ZHIP), University of Zurich, Zurich, Switzerland; 3 Biomedical Research Center, Zhongshan Hospital, Fudan University, Shanghai, China; Goethe Universität Frankfurt, Germany

## Abstract

**Background:**

The aging gene p66^Shc^, is an important mediator of oxidative stress-induced vascular dysfunction and disease. In cultured human aortic endothelial cells (HAEC), p66^Shc^ deletion increases endothelial nitric oxide synthase (eNOS) expression and nitric oxide (NO) bioavailability via protein kinase B. However, the putative role of the NO pathway on p66^Shc^ activation remains unclear. This study was designed to elucidate the regulatory role of the eNOS/NO pathway on p66^Shc^ activation.

**Methods and Results:**

Incubation of HAEC with oxidized low density lipoprotein (oxLDL) led to phosphorylation of p66^Shc^ at Ser-36, resulting in an enhanced production of superoxide anion (O_2_^-^). In the absence of oxLDL, inhibition of eNOS by small interfering RNA or L-NAME, induced p66^Shc^ phosphorylation, suggesting that basal NO production inhibits O_2_^-^ production. oxLDL-induced, p66^Shc^-mediated O_2_- was prevented by eNOS inhibition, suggesting that when cells are stimulated with oxLDL eNOS is a source of reactive oxygen species. Endogenous or exogenous NO donors, prevented p66^Shc^ activation and reduced O_2-_ production. Treatment with tetrahydrobiopterin, an eNOS cofactor, restored eNOS uncoupling, prevented p66^Shc^ activation, and reduced O_2_- generation. However, late treatment with tetrahydropterin did not yield the same result suggesting that eNOS uncoupling is the primary source of reactive oxygen species.

**Conclusions:**

The present study reports that in primary cultured HAEC treated with oxLDL, p66^Shc^-mediated oxidative stress is derived from eNOS uncoupling. This finding contributes novel information on the mechanisms of p66^Shc^ activation and its dual interaction with eNOS underscoring the importance eNOS uncoupling as a putative antioxidant therapeutical target in endothelial dysfunction as observed in cardiovascular disease.

## Introduction

Endothelial nitric oxide synthase (eNOS) produces nitric oxide (NO), a key factor involved in maintaining endothelial homeostasis [1]. Further, NO plays a key role in preventing endothelial dysfunction by scavenging O_2_- [2], reducing adhesion of platelets and leukocytes [3], and inhibiting migration and proliferation of smooth muscle cells [4]. However, under pathological conditions eNOS can become a source of reactive oxygen species [5], [6], [7]. The underlying mechanisms of this switch include oxidization of tetrahydrobiopterin [5], depletion of tetrahydrobiopterin [8], and dephosphorylation of eNOS at Thr495 [9].

The p66^Shc^ adaptor protein is an important mediator of oxidative stress-induced vascular dysfunction [10], acting as a redox enzyme implicated in mitochondrial ROS generation and the translation of oxidative signals into apoptosis [11], [12], [13], [14], [15]. Genetic deletion of p66^Shc^ in the mouse extends lifespan by reducing the production of intracellular oxidants [16] and in ApoE^−/−^ mice treated with high fat diet limits atherosclerotic plaque formation due to decreased lipid peroxidation [17]. Previous studies reported that in human aortic endothelial cells oxidized LDL increases ROS production via phosphorylation of the p66^Shc^ protein at ser36 through the lectin-like oxLDL receptor-1, activation of protein kinase C beta-2, and c-Jun N-terminal kinase [18]; of note, this effect can be prevented by p66^Shc^ silencing. These results underscored the critical role of p66^Shc^ in oxLDL-induced oxidative stress in endothelial cells [18]. Indeed, activation of p66^Shc^ leads to a surge of reactive oxygen species from mitochondria [16], [19] and/or via NADPH oxidase [18], [20].

Further, it has been reported that p66^Shc^ overexpression inhibits eNOS-dependent NO production [21], while deletion of p66^Shc^ leads to increased phosphorylation of eNOS at the activatory phosphorylation site Ser1177 through the protein kinase B pathway [22]. These findings imply an important role of p66^Shc^ adaptor protein in modulating endothelium-derived NO production [22]. On the other hand, the role of endothelium-derived NO in controlling p66^Shc^ activation remained not known. The present study was therefore designed to study the effects of eNOS, as well as NO, on the expression of the p66^Shc^ adaptor protein.

## Materials and Methods

### Cell culture experiments

Primary human aortic endothelial cells (HAEC; Clonetics, Allschwil, Switzerland), from passage 4 to 6, were used. The cells were cultured and passaged in EBM-2 medium supplied with EGM-2 bulletkit (Clonetics, Walkersville USA). Experiments were performed in EBM medium with 0.5% FBS. Cells were harvested for further measurements (Western blotting, superoxide production measurement) either within 60 minutes or after 24 hours of exposure to oxLDL. NO donors or inhibitors were added to medium 60 minutes before exposure of the cells to oxLDL. Tetrahydrobiopterin was added to the medium 60 minutes prior to (Before, B), 45 minutes (After Early, AE) or 16 hours (After Later, AL) after the oxLDL stimulation.

### Materials

Apocynin was purchased from SAFC (Saint. Louis, MO, USA). 8-Bromoguanosine 3′,5′-cyclic monophosphate, bradykinin, calcium ionophore (A23187), N (G)-nitro-L- arginine methyl ester (L-NAME), ODQ, tetrahydrobiopterin, siRNA against eNOS and N-TER Nanoparticle siRNA Transfection system, and anti-α-tubulin antibody were obtained from Sigma (Saint Louis, MO, USA). Anti-Shc/p66 (pSer36) antibody was purchased from Calbiochem (Darmstadt, Germany). DETA NONOate, DEA NONOate, and KT5823 were purchased from Cayman (Michigan, USA). Oxidized LDL and LDL are bought from Biomedical Technologies (Stoughton, MA, USA). Anti-Shc antibody was purchased from Cell Signaling (Danvers, MA, USA). Anti-eNOS antibody was bought from B&D transduction laboratories (NJ, USA). Anti-rabbit and Anti-mouse second antibodies were bought from GE healthcare (Buckinghamshire, UK).

### Measurement of reactive oxygen species

O_2_^-^ generation in intact cells was assessed using the spin trap 1-hydroxy-3-methoxycarbonyl-2,2,5,5-tetramethyl-pyrrolidine (CMH). Human aortic endothelial cells were collected in Krebs-HEPES solution containing diethyldithiocarbonic acid sodium salt (DETC 5 uM), deferoxamine (25 uM), and CMH (200 µM). The formation of the stable spin label 3-methoxycarbonyl-proxyl (CM') was determined at room temperature with an EMX ESR spectrometer (Bruker, Bremen Germany).

### Western blotting

Protein expression was determined by Western blot analysis. Samples from cell culture were collected in lysis buffer (NaCl 150 mM, EDTA 1 mM, NaF 1 mM, DTT 1 mM, aprotinin 10 µg/µl, leupeptin 10 µg/µl, Na_3_VO_4_ 0.1 mM, PMSF 1 mM, and NP-40 0.5%). Proteins were loaded on a separating gel (SDS-PAGE) and transferred to a polyvinylidene fluoride membrane by semidry transfer. The membranes were incubated with antibody. Related signals were quantified using a Scion image software (Scion Corporation, Frederick, Maryland, USA).

### Small interfering RNA (siRNA)

In certain experiments, predesigned small interfering RNA (siRNA) against eNOS (5′-CCUACAUCUGCAACCACAU[dT][dT]-3′; 10 nM) (Sigma, Saint Louis, MO, USA) were applied. HAEC were transfected with siRNA against eNOS at final concentration of 10 nM in a serum-free medium using N-TER Nanoparticle siRNA Transfection System (NFS, Sigma, Saint Louis, MO, USA), according to the manufacture's protocol. Cells were incubated with siRNA in serum-free and antibiotics-free medium for four hours, followed by normal growth medium for another 24 hours prior to the experiments. Nanoparticle Formation Solution (NFS) and scrambled siGAPDH (5′-GGUUUACAUGUUCCAAUAU[dT][dT]-3′; 10 nM) were used as negative controls.

### Data analysis

Data are presented as means±SEM, Statistical analysis was performed by one–way ANOVA followed by a *post hoc* comparison using the Bonferroni test (Prism, GraphPad Software, San Diego, CA, USA). Differences were considered to indicate statistically significant when the P value was less than 0.05.

## Results

### 1. oxLDL induces p66^Shc^ adaptor protein phosphorylation and eNOS uncoupling

**Figure 1:**
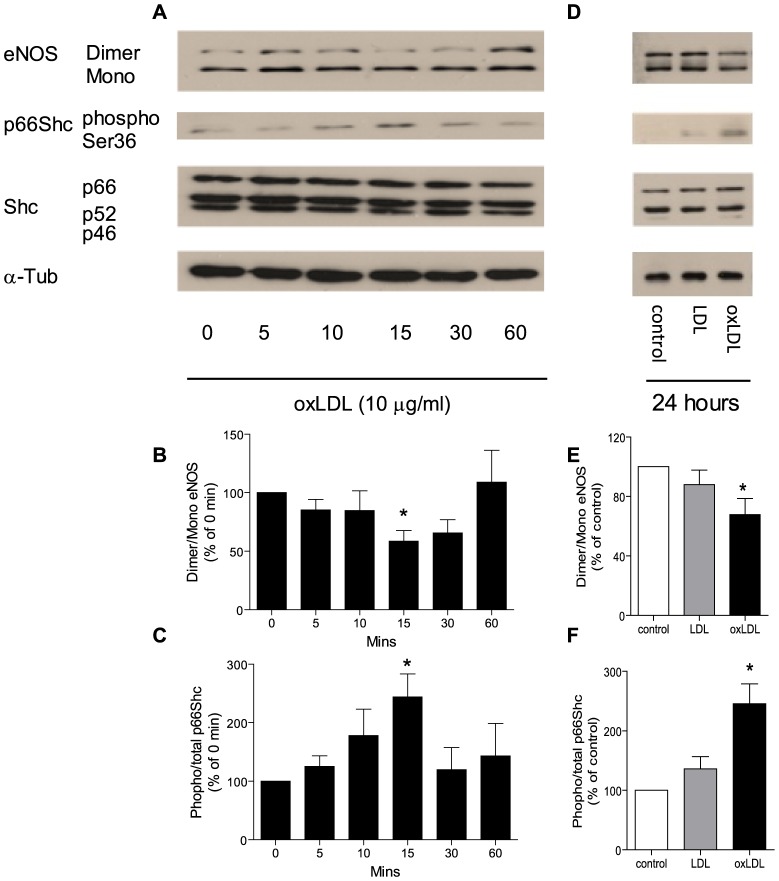
Representive Western blot (A, D) and densitometric quantification of eNOS uncoupling (B, E) and phospho-p66^Shc^ protein (C, F) expression in HAEC treated with oxLDL within sixty minutes (A, B, C) or for twenty four hours (D, E, F). eNOS uncoupling was presented as ratio of dimer/monomer form of eNOS. The phosphorylation of p66^Shc^ was normalized to total p66^Shc^ protein and total p66^Shc^ was normalized to α-tubulin. Results are presented as means±SEM; n = 6. * p<0.05 vs. cells at 0 minutes or cells under control condition.

In HAEC, incubation with oxLDL (10 µg/ml) induced a transient phosphorylation of p66^Shc^ at the Ser-36 amino acid residue. Nonetheless, total protein levels of p66^Shc^, as well as that of the other two isoforms of the Shc adaptor protein family, p52^Shc^ and p46^Shc^, did not change within 60 minutes. In parallel, oxLDL transiently reduced the dimer/monomor ratio of eNOS within 60 minutes (**Figure 1 A, B, and C**).

In line with our previous findings [18], phosphorylation of p66^Shc^ was also detectable after 24 hours of incubation of the cells with oxLDL (10 µg/ml). After 24 hours, eNOS dimer/monomor ratio was once again reduced, while compared to cells under control condition or treated with native LDL (10 µg/ml), denoting a biphasic response (**Figure 1 D, E, and F**).

### 2. eNOS plays a dual role for p66^Shc^ phosphorylation

#### 2.1 Inhibition of eNOS enhances p66^Shc^ phosphorylation under basal condition, but reduces p66^Shc^ phosphorylation under stimulated condition

**Figure 2:**
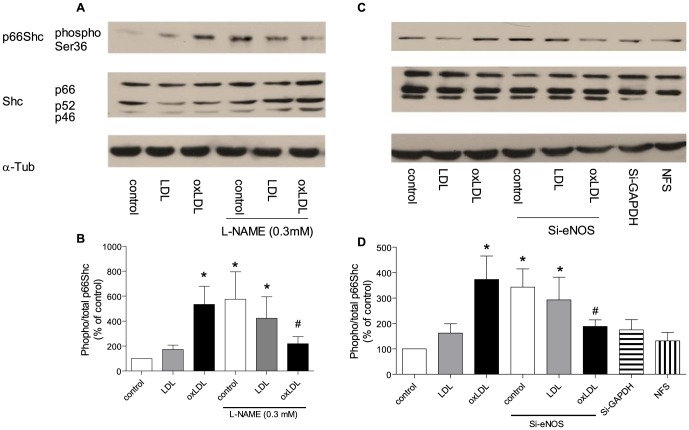
Representive Western blot (A, C) and densitometric quantification of phospho-p66^Shc^ protein expression (B, D) in HAEC after twenty-four hours of incubation with oxLDL in the presence of nitric oxide synthase inhibitor (L-NAME 0.3 mM; A and B) and in the presence of eNOS siRNA (10 nM, C and D). The phosphorylation of p66^Shc^ was normalized to total p66^Shc^ protein and total p66^Shc^ was normalized to α-tubulin. Results are presented as means±SEM; n = 8. * p<0.05 vs. cells under control conditions. # p<0.05 vs. oxLDL alone. (NFS: nanoparticle formation solution).

After 24 hours of exposure to oxLDL, p66^Shc^ phosphorylation was increased in cells treated with L-NAME or L-NAME combined with native LDL. However in the presence of L-NAME, oxLDL-induced p66^Shc^ phosphorylation was significantly reduced (**Figure 2 A, B**).

To further corroborate the findings with L-NAME treatment, small interfering RNA against eNOS (Si-eNOS, Figure S1) were used. In line with the pharmacologic inhibition of eNOS, siRNA induced a significantly higher level of phosphorylated p66^Shc^ under quiescent condition. Furthermore, phosphorylation of p66^Shc^ was reduced when cells were exposed to both oxLDL and Si-eNOS (**Figure 2 C, D**).

#### 2.2 Activation of Nitric oxide pathway prevents p66^Shc^ phosphorylation

**Figure 3:**
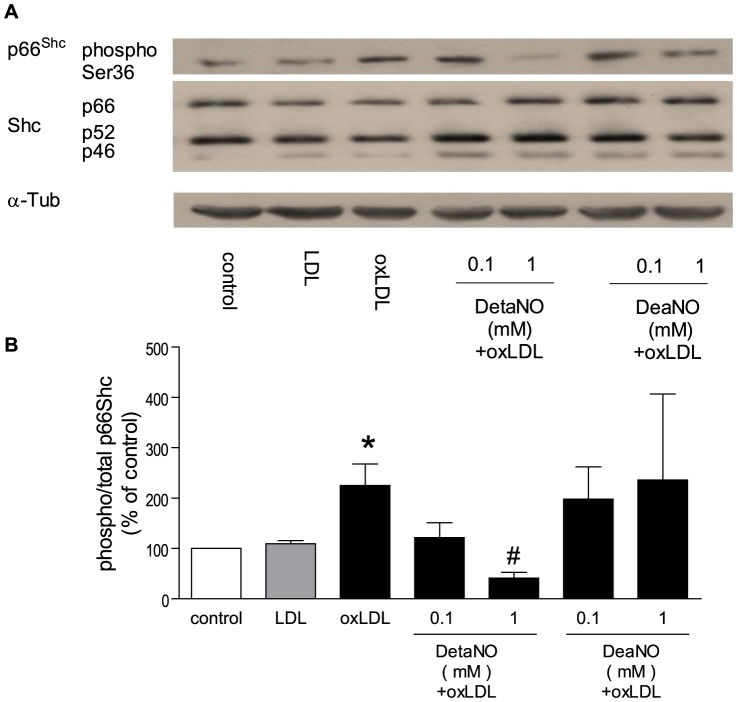
Representative Western blot (A) and densitometric quantification of phospho-p66^Shc^ protein (B) expression in HAEC after twenty-four hours of incubation with oxLDL in the presence of nitric oxide donor (DetaNO 0.1-1 mM or DeaNO 0.1–1 mM). The phosphorylation of p66^Shc^ was normalized to total p66^Shc^ protein and total p66^Shc^ was normalized to α-tubulin. Results are presented as means±SEM; n = 5. * p<0.05 vs. cells under control conditions. # p<0.05 vs. oxLDL alone.

**Figure 4:**
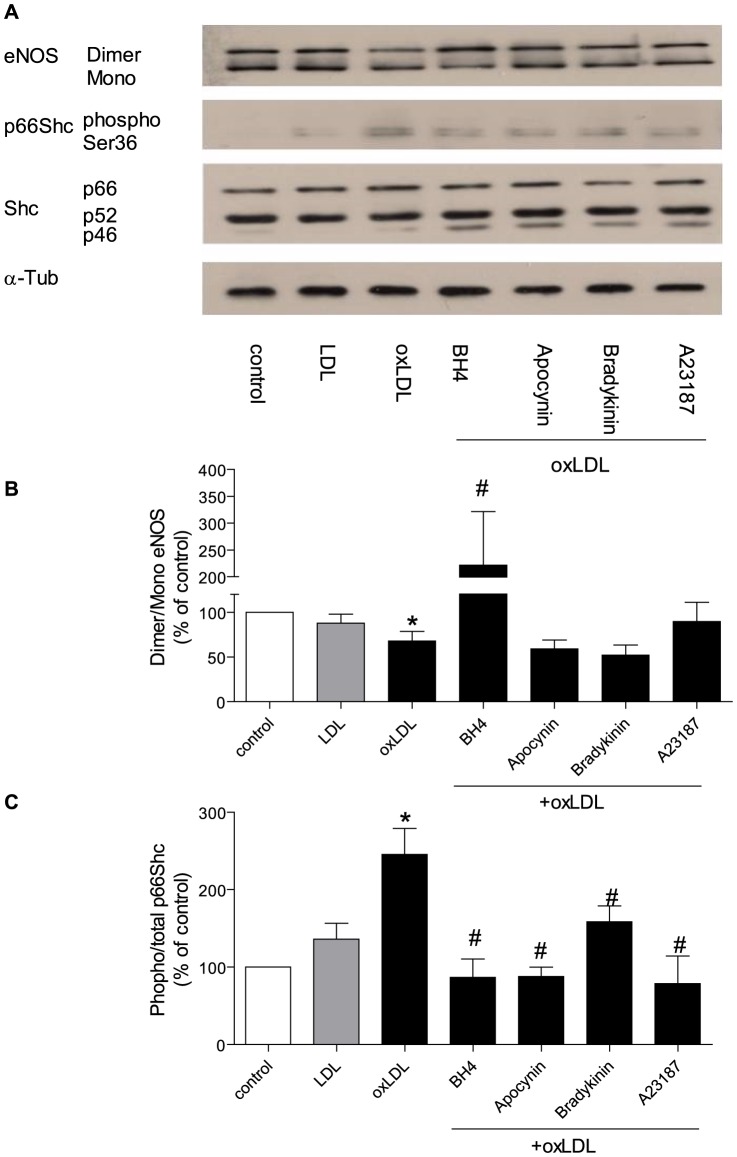
Representive Western blot (A) and densitometric quantification of eNOS uncoupling (B) and phospho-p66Shc protein (C) expression in HAEC after twenty-four hours incubation with oxLDL in the presence of tetrahydrobiopterin (BH4 10 µM), apocynin (100 µM), bradykinin (1 µM) or calcium ionophore (A23187 1 µM). eNOS uncoupling was presented as ratio of dimer/monomer form of eNOS. The phosphorylation of p66^Shc^ was normalized to total p66^Shc^ protein and total p66^Shc^ was normalized to α-tubulin. Results are presented as means±SEM; n = 8. * p<0.05 vs. cells under control conditions. # p<0.05 vs. oxLDL alone.

After 24 hours, DetaNO [23], a NO donor with a half life of up to 20 hours, at 1 mM, but not at 0.1 mM, significantly reduced oxLDL-induced p66^Shc^ phosphorylation. DeaNO [24], another NO donor which has a half life of 2 minutes, either at 1 mM or 0.1 mM, did not significantly change the level of p66^Shc^ phosphorylation (**Figure 3**). After 24 hours, bradykinin (1 µM) [25] or calcium ionphore (1 µM) [26] significantly reduced oxLDL-induced p66^Shc^ phosporylation (**Figure 4**).

8-Br-cGMP (1 mM) [27], an analogue of cyclic guanosine monophosphate, prevented the oxLDL-induced p66^Shc^ phosphorylation after 24 hours stimulation of oxLDL (Figure S2).

#### 2.3 Inhibition of protein kinase G pathway does not change p66^Shc^ phosphorylation

ODQ (10 µM) [28], an inhibitor of soluble guanylyl cyclase, did not significantly change the phosphorylation level of p66^Shc^ protein, either in the presence or absence of oxLDL. Likewise, KT5823 (1 µM) [29], an inhibitor of protein kinase G, did not significantly alter the phosphorylation level of the p66^Shc^ protein, either in the presence or absence of oxLDL (Figure S3).

### 3. Tetrahydrobiopterin prevents p66^Shc^ phosphorylation and restores eNOS uncoupling

After 24 h of exposure to oxLDL, tetrahydrobiopterin (10 µM) [30], a cofactor of eNOS, increased the dimer/monomor ratio of eNOS and prevented p66^Shc^ phosphorylation. Furthermore, apocynin, an antioxidant, significantly reduced the phosphorylation level of p66^Shc^, but did not change the dimer/monomor ratio of eNOS (**Figure 4**)

**Figure 5:**
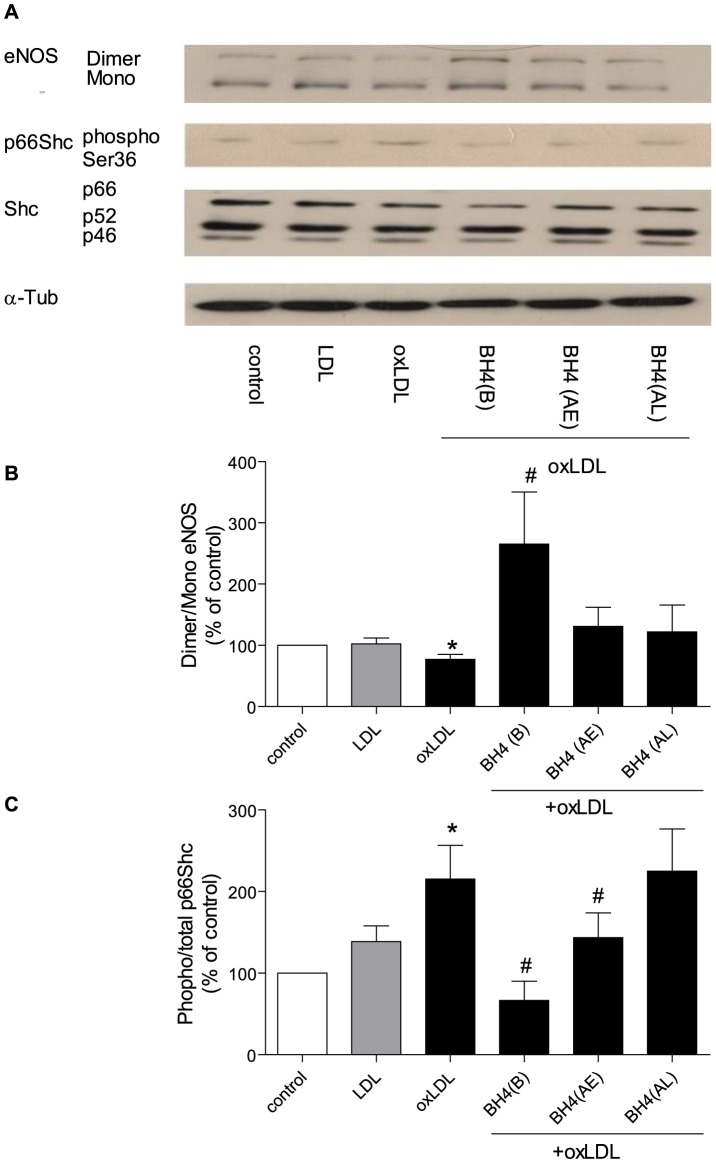
Representive Western blot (A) and densitometric quantification of eNOS uncoupling (B) and phospho-p66^Shc^ protein (C) in HAEC after twenty-four hours incubation with oxLDL in the presence of tetrahydrobiopterin [BH4 10 µM; before (B), forty five minutes after (After Early, AE), or sixteen hours after (After Later, AL) oxLDL treatment]. eNOS uncoupling was presented as ratio of dimer/monomer form of eNOS. The phosphorylation of p66^Shc^ was normalized to total p66^Shc^ protein and total p66^Shc^ was normalized to α-tubulin. Results are presented as means±SEM; n = 8. * p<0.05 vs. cells treated with oxLDL alone.

Under the same experimental conditions, administration of tetrahydrobiopterin (45 minutes after oxLDL), reduced p66^Shc^ phosphorylation, but did not change the eNOS dimer/monomor ratio. Administration of tetradydrobiopterin (16 hours after oxLDL), did not change the eNOS dimer/monomor ratio nor oxLDL-induced p66^Shc^ phosphorylation (**Figure 5**).

### 4. oxLDL induces reactive oxygen species

**Figure 6:**
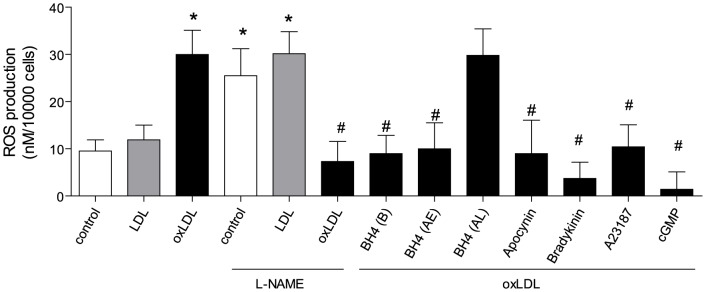
O_2_- production after twenty-four hours of incubation with oxLDL in the presence or absence of tetrahydrobiopterin [BH4 10 µM, before (B), forty five minutes after (AE), and sixteen hours after (AL) oxLDL treatment], apocynin (100 µM), bradykinin (1 µM), calcium ionophore (1 µM), L-NAME (0.3 mM) and cGMP (1 mM). Results are presented as means±SEM; n = 8. * p<0.05 vs. cells under control conditions. # p<0.05 vs. oxLDL alone.

The production of reactive oxygen species was measured in HAEC 24 hours after exposure to oxLDL. In the presence of oxLDL, but not native LDL, endothelial cells exhibited an increased production of O_2_-. This enhanced production of O_2_- was inhibited by apocynin (100 µM) [18]. In the absence of oxLDL, L-NAME alone induced a high level of O_2_- in endothelial cells, whereas in the presence of oxLDL, L-NAME significantly reduced the production of O_2_^-^ (**Figure 6**).

Bradykinin or calcium ionophore significantly reduced oxLDL-induced O_2_^-^ production. 8-Br-cGMP reduced the level of oxLDL-induced O_2_^-^ (**Figure 6**).

Tetrahydrobiopterin, administrated either 60 minutes before (B) or 45 minutes after oxLDL stimulation (AE), significantly reduced oxLDL-induced O_2_^-^ production. However the late administration (AL) failed to decrease O_2_^-^ production in endothelial cell (**Figure 6**).

oxLDL did not change levels of protein peroxynitrition in endothelial cells (Figure S4).

## Discussion

This study analyzed the molecular mechanisms underlying the dual role of eNOS and its product NO in controlling the activation of p66^Shc^ adaptor protein – an important mediator of ROS-dependent cardiovascular disease. In primary HAECs, inhibition of eNOS induced p66^Shc^ activation and ROS production, suggesting that under basal condition eNOS provides an inhibitory signal preventing p66^Shc^ activation and p66^Shc^-dependent ROS production. In contrast, in primary HAECs stimulated by oxLDL, eNOS uncoupled and acted as the primary source of p66^Shc^-mediated ROS. Accordingly, under these conditions tetrahydrobiopterin restored eNOS coupling and function, prevented p66^Shc^ activation, and reduced superoxide generation.

p66^Shc^ adaptor protein is importantly involved in various forms of cardiovascular disease by providing reactive oxygen species. Of note, genetic deletion of p66^Shc^ protein preserves endothelial function in aging mice and extends life span of these animals. Furthermore, p66^Shc^ deletion preserves endothelial function and reduces plaque formation in ApoE^−/−^ mice by virtue of a reduced production of reactive oxygen species [16], [17]. Similar effects occur in diabetic endothelial dysfunction and in experimental stroke [11], [31].

p66^Shc^ phosphorylation, at serine 36 amino acid residue, mediates ROS production in different settings [16], [18], [32] via activation of NADPH oxidase [18], [20] and/or release of ROS from mitochondria via opening of PTC pores [33].

Interestingly, in unstimulated HAECs, inhibition of eNOS by the pharmacological inhibitor LNAME or by using siRNA preventing translation of the protein, both induced p66^Shc^ phosphorylation and increased generation of O_2_-, suggesting that under these conditions, basal release of NO inhibits the activation of the adaptor protein and the formation of ROS, thereby playing a so far unrecognized antioxidant role. Importantly, neither cyclic GMP nor protein kinase G is involved in this process since this effect was not observed after treatment with ODQ or KT5823, suggesting a direct interaction between O_2_^-^ and NO in the present set up [2].

oxLDL, a key mediator of atherosclerosis, induced p66^Shc^ phosphorylation and in turn stimulated O_2_^-^ production in endothelial cells, confirming that the adaptor protein p66^Shc^ is activated by the modified lipoprotein and a crucial regulator of intracellular ROS generation [16], [18]. In the presence of oxLDL, the NO donor (DetaNO) as well as receptor-operated activators of eNOS such as bradykinin or receptor-independent activators such as calcium ionophore, reduced p66^Shc^ activation and decreased O_2_^-^ production. These results suggest that NO also provides a protective effect against reactive oxygen species under stimulated condition. Treatment with cyclic GMP prevented p66^Shc^ phosphorylation and superoxide generation after 24 hours, indicating that the protective role of basal NO is mediated through the NO-cGMP pathway [1], [34].

Of note, oxLDL transiently induced eNOS uncoupling and p66^Shc^ phosphorylation within 15 minutes of incubation. After 24 h of stimulation with oxLDL, inhibition of eNOS reduced p66^Shc^ phosphorylation and decreased the O_2_^-^ production suggesting that under stimulated conditions eNOS becomes a source of reactive oxygen species [5], [35]. Apocynin reduced oxLDL-induced p66^Shc^ phosphorylation and superoxide production, but did not restore eNOS uncoupling, indicating that eNOS uncoupling is upstream of p66^Shc^ activation. In line with that, tetrahydrobiopterin, a cofactor of eNOS, restored oxLDL-induced eNOS uncoupling and p66^Shc^-dependent superoxide generation, once again suggesting that eNOS uncoupling is the primary source of oxLDL-induced, p66^Shc^-mediated reactive oxygen species.

Uncoupling of eNOS is an important mechanism of endothelial dysfunction in atherosclerosis [36], diabetes [37], and hypertension [5]. The underlying mechanisms of eNOS uncoupling include tetrahydrobiopterin deficiency [38], decreased levels of L-arginine [39], enhanced levels of asymmetric dimethylarginine [40] or S-glutathionylation of eNOS [41]. Deficiency of tetrahydrobiopterin seems to be the primary cause for eNOS uncoupling under pathophysiological conditions [42]. Tetrahydrobiopterin facilitates electron transfer from the eNOS reductase domain and maintains the heme prosthetic group in its redox active forms. Further tetrahydrobiopterin promotes and stabilizes eNOS protein monomers into the active homodimeric form of the enzyme [38], [43]. Increased levels of tetrahydrobiopterin enhance eNOS activity in cultured cells [44], [45] and promote vasodilatation in isolated mouse pial arterioles [46]. In animal experiments, tetrahydrobiopterin treatment reduces oxidative stress and preserves endothelial function in streptozotocin-induced type I diabetes [47], insulin-resistant type II diabetes [48], DOCA-salt induced hypertension [49] and ischemia/reperfusion induced injury [50]. However, results in human studies are controversial; it has been reported that tetrahydrobiopterin improves endothelial dysfunction in postmenopausal women [51], subjects with hypercholesterolemia [52], patients with chronic coronary disease [53], smokers [54] and type II diabetic patients [55]. Additionally, it was reported that oral tetrahydrobiopterin does not alter vascular redox state or endothelial function owing to systemic and vascular oxidation of tetrahydrobiopterin [56]. In the present study, tetrahydrobiopterin treatment, prior to oxLDL stimulation, prevented p66^Shc^-mediated oxidative stress, confirming that tetrahydrobiopterin confers a protective effect on eNOS coupling. Interestingly, this effect was also observed with tetrahydrobiopterin treatment early after oxLDL stimulation, but not in the late treatment, implying that other sources of oxidative stress participate in ROS generation at late stage, which cannot be inhibited by a late tetrahydrobiopterin treatment. p66^Shc^ adaptor protein is reported to translates oxidative damage into cell death by acting as mediator of reactive oxygen species within mitochondria [33]. We reported previously that upon oxLDL stimulation in human aortic endothelial cells, p66^Shc^ protein is activated leading to increased p47phox protein expression and superoxide anion production; this effect is mediated via lectin-like oxLDL receptor-1, activation of protein kinase C beta-2 and c-Jun N-terminal kinase, respectively [18]. Interestingly,, this effect could not be prevented by p66^Shc^ silencing. [18]. Thus the results in the present study provide a possible molecular explanation for those antioxidant treatments in large, long-term clinic trials, which have failed to improve cardiovascular outcome [57], [58], [59].

**Figure 7:**
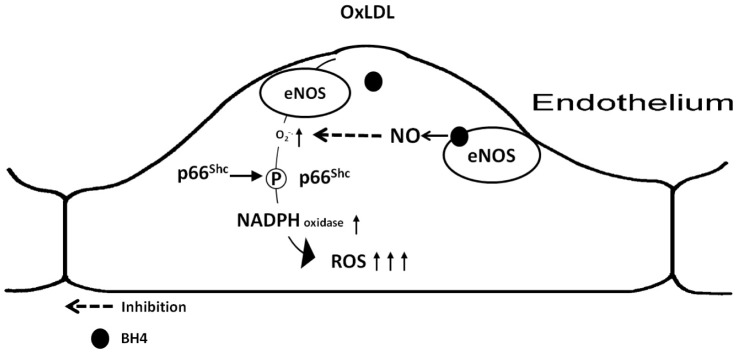
Putative role of eNOS in oxLDL-induced, p66Shc- mediated oxidative stress in HAEC. eNOS uncoupling is the primary source of oxLDL-induced oxidative stress in endothelial cells, leading to the p66Shc activation and later surge of ROS production. Supply with nitric oxide or reversal eNOS uncoupling reduces p66Shc activation and ROS production.

The present experiments performed in cultured human primary endothelial cells describe a dual role of eNOS for p66^Shc^ protein activation and reactive oxygen species generation. It appears that eNOS uncoupling is a crucial player in oxLDL-induced and p66^Shc^-mediated intracellular reactive oxygen species generation (**Figure 7**). These findings provide important mechanistic information about endothelial dysfunction, thus eNOS uncoupling represents a potential therapeutic target for early intervention of atherosclerosis.

## Supporting Information

Figure S1 Representative Western blot of eNOS, total Shc protein (p66, p52, and p46), and α-tubulin expression in HAEC treated with small interfering RNA against eNOS. (SiGAPDH: small interfering RNA against GAPDH; NFS: nanoparticle formation solution).
(EPS)

Figure S2 Representative Western blot (A) and densitometric quantification of phospho-p66^Shc^ protein expression (B) in HAEC after twenty-four hours of incubation with oxLDL in the presence of cGMP (1 mM) (A). The phosphorylation of p66^Shc^ was normalized to total p66^Shc^ protein and total p66^Shc^ was normalized to α-tubulin. Results are presented as means±SEM; n = 5. * p<0.05 vs. cells under control conditions. # p<0.05 vs. oxLDL alone.
(EPS)

Figure S3 Densitometric quantification of phospho-p66^Shc^ protein expression in HAEC after twenty-four hours of incubation with oxLDL in the presence or absence of KT 5832 (1 µM). The phosphorylation of p66^Shc^ was normalized to total p66^Shc^ protein and total p66^Shc^ was normalized to α-tubulin. Results are presented as means±SEM; n = 5. * p<0.05 vs. cells under control conditions.
(EPS)

Figure S4 Densitometric quantification of protein peroxynitrition in HAEC after twenty-four hours of incubation with oxLDL. The level of protein peroxynitrition was normalized to α-tubulin. Results are presented as means±SEM; n = 5.
(EPS)
